# Supplementary material for: A Critical View of the Application of the APEX Software (Aqueous Photochemistry of Environmentally-Occurring Xenobiotics) to Predict Photoreaction Kinetics in Surface Freshwaters
Source: Molecules. 2019 Dec 18;25(1):9. doi: 10.3390/molecules25010009 (PMC7017383; doi:10.3390/molecules25010009)
Supplement: Supplementary file 1 [file molecules-25-00009-s001.zip › 3.2.4_gcc-4.4.0.zip!/3.2.4_gcc-4.4.0/3.2.4_gcc-4.4.0/doc/octave/html/interpreter/Using-Octave-Mode.html]

Using Octave Mode - Untitled


Next: Running Octave From Within Emacs,
Previous: Installing EOS,
Up: Emacs Octave Support


---

### G.2 Using Octave Mode

If you are lucky, your sysadmins have already arranged everything so
that Emacs automatically goes into Octave mode whenever you visit an
Octave code file as characterized by its extension .m. If not,
proceed as follows.

1. To begin using Octave mode for all .m files you visit, add the
   following lines to a file loaded by Emacs at startup time, typically
   your ~/.emacs file:

   ```
             (autoload 'octave-mode "octave-mod" nil t)
             (setq auto-mode-alist
                   (cons '("\\.m$" . octave-mode) auto-mode-alist))
   ```

   - Finally, to turn on the abbrevs, auto-fill and font-lock features
     automatically, also add the following lines to one of the Emacs startup
     files:

     ```
               (add-hook 'octave-mode-hook
                         (lambda ()
                           (abbrev-mode 1)
                           (auto-fill-mode 1)
                           (if (eq window-system 'x)
                               (font-lock-mode 1))))
     ```

     See the Emacs manual for more information about how to customize
     Font-lock mode.

In Octave mode, the following special Emacs commands can be used in
addition to the standard Emacs commands.

`C-h m`: Describe the features of Octave mode. `LFD`: Reindent the current Octave line, insert a newline and indent the new line (`octave-reindent-then-newline-and-indent`). An abbrev before point is expanded if `abbrev-mode` is non-`nil`. `TAB`: Indents current Octave line based on its contents and on previous lines (`indent-according-to-mode`). `;`: Insert an “electric” semicolon (`octave-electric-semi`). If `octave-auto-indent` is non-`nil`, reindent the current line. If `octave-auto-newline` is non-`nil`, automagically insert a newline and indent the new line. `` ` ``: Start entering an abbreviation (`octave-abbrev-start`). If Abbrev mode is turned on, typing `` `C-h `` or `` `? `` lists all abbrevs. Any other key combination is executed normally. Note that all Octave abbrevs start with a grave accent. `M-LFD`: Break line at point and insert continuation marker and alignment (`octave-split-line`). `M-TAB`: Perform completion on Octave symbol preceding point, comparing that symbol against Octave's reserved words and built-in variables (`octave-complete-symbol`). `M-C-a`: Move backward to the beginning of a function (`octave-beginning-of-defun`). With prefix argument N, do it that many times if N is positive; otherwise, move forward to the N-th following beginning of a function. `M-C-e`: Move forward to the end of a function (`octave-end-of-defun`). With prefix argument N, do it that many times if N is positive; otherwise, move back to the N-th preceding end of a function. `M-C-h`: Puts point at beginning and mark at the end of the current Octave function, i.e., the one containing point or following point (`octave-mark-defun`). `M-C-q`: Properly indents the Octave function which contains point (`octave-indent-defun`). `M-;`: If there is no comment already on this line, create a code-level comment (started by two comment characters) if the line is empty, or an in-line comment (started by one comment character) otherwise (`octave-indent-for-comment`). Point is left after the start of the comment which is properly aligned. `C-c ;`: Puts the comment character # (more precisely, the string value of `octave-comment-start`) at the beginning of every line in the region (`octave-comment-region`). With just `C-u` prefix argument, uncomment each line in the region. A numeric prefix argument N means use N comment characters. `C-c :`: Uncomments every line in the region (`octave-uncomment-region`). `C-c C-p`: Move one line of Octave code backward, skipping empty and comment lines (`octave-previous-code-line`). With numeric prefix argument N, move that many code lines backward (forward if N is negative). `C-c C-n`: Move one line of Octave code forward, skipping empty and comment lines (`octave-next-code-line`). With numeric prefix argument N, move that many code lines forward (backward if N is negative). `C-c C-a`: Move to the `real' beginning of the current line (`octave-beginning-of-line`). If point is in an empty or comment line, simply go to its beginning; otherwise, move backwards to the beginning of the first code line which is not inside a continuation statement, i.e., which does not follow a code line ending in ... or \, or is inside an open parenthesis list. `C-c C-e`: Move to the `real' end of the current line (`octave-end-of-line`). If point is in a code line, move forward to the end of the first Octave code line which does not end in ... or \ or is inside an open parenthesis list. Otherwise, simply go to the end of the current line. `C-c M-C-n`: Move forward across one balanced begin-end block of Octave code (`octave-forward-block`). With numeric prefix argument N, move forward across n such blocks (backward if N is negative). `C-c M-C-p`: Move back across one balanced begin-end block of Octave code (`octave-backward-block`). With numeric prefix argument N, move backward across N such blocks (forward if N is negative). `C-c M-C-d`: Move forward down one begin-end block level of Octave code (`octave-down-block`). With numeric prefix argument, do it that many times; a negative argument means move backward, but still go down one level. `C-c M-C-u`: Move backward out of one begin-end block level of Octave code (`octave-backward-up-block`). With numeric prefix argument, do it that many times; a negative argument means move forward, but still to a less deep spot. `C-c M-C-h`: Put point at the beginning of this block, mark at the end (`octave-mark-block`). The block marked is the one that contains point or follows point. `C-c ]`: Close the current block on a separate line (`octave-close-block`). An error is signaled if no block to close is found. `C-c f`: Insert a function skeleton, prompting for the function's name, arguments and return values which have to be entered without parentheses (`octave-insert-defun`). `C-c C-h`: Search the function, operator and variable indices of all info files with documentation for Octave for entries (`octave-help`). If used interactively, the entry is prompted for with completion. If multiple matches are found, one can cycle through them using the standard , (`Info-index-next`) command of the Info reader. The variable `octave-help-files` is a list of files to search through and defaults to `'("octave")`. If there is also an Octave Local Guide with corresponding info file, say, octave-LG, you can have `octave-help` search both files by ``` (setq octave-help-files '("octave" "octave-LG")) ``` in one of your Emacs startup files.

A common problem is that the <RET> key does *not* indent the
line to where the new text should go after inserting the newline. This
is because the standard Emacs convention is that <RET> (aka
`C-m`) just adds a newline, whereas <LFD> (aka `C-j`) adds a
newline and indents it. This is particularly inconvenient for users with
keyboards which do not have a special <LFD> key at all; in such
cases, it is typically more convenient to use <RET> as the <LFD>
key (rather than typing `C-j`).

You can make <RET> do this by adding

```
     (define-key octave-mode-map "\C-m"
       'octave-reindent-then-newline-and-indent)
```

to one of your Emacs startup files. Another, more generally applicable
solution is

```
     (defun RET-behaves-as-LFD ()
       (let ((x (key-binding "\C-j")))
         (local-set-key "\C-m" x)))
     (add-hook 'octave-mode-hook 'RET-behaves-as-LFD)
```

(this works for all modes by adding to the startup hooks, without having
to know the particular binding of <RET> in that mode!). Similar
considerations apply for using <M-RET> as <M-LFD>. As Barry
A. Warsaw <bwarsaw@cnri.reston.va.us> says in the documentation for his
`cc-mode`, “This is a very common question. `:-)` If you want
this to be the default behavior, don't lobby me, lobby RMS!”

The following variables can be used to customize Octave mode.

`octave-auto-indent`: Non-`nil` means auto-indent the current line after a semicolon or space. Default is `nil`. `octave-auto-newline`: Non-`nil` means auto-insert a newline and indent after semicolons are typed. The default value is `nil`. `octave-blink-matching-block`: Non-`nil` means show matching begin of block when inserting a space, newline or ; after an else or end keyword. Default is `t`. This is an extremely useful feature for automatically verifying that the keywords match—if they don't, an error message is displayed. `octave-block-offset`: Extra indentation applied to statements in block structures. Default is 2. `octave-continuation-offset`: Extra indentation applied to Octave continuation lines. Default is 4. `octave-continuation-string`: String used for Octave continuation lines. Normally \. `octave-mode-startup-message`: If `t` (default), a startup message is displayed when Octave mode is called.

If Font Lock mode is enabled, Octave mode will display

- strings in `font-lock-string-face`- comments in `font-lock-comment-face`- the Octave reserved words (such as all block keywords) and the text
      functions (such as cd or who) which are also reserved
      using `font-lock-keyword-face`- the built-in operators (&&, ==, ...) using
        `font-lock-reference-face`- and the function names in function declarations in
          `font-lock-function-name-face`.

There is also rudimentary support for Imenu (currently, function names
can be indexed).

You can generate TAGS files for Emacs from Octave .m files using
the shell script `octave-tags` that is installed alongside your copy of
Octave.

Customization of Octave mode can be performed by modification of the
variable `octave-mode-hook`. If the value of this variable is
non-`nil`, turning on Octave mode calls its value.

If you discover a problem with Octave mode, you can conveniently send a
bug report using `C-c C-b` (`octave-submit-bug-report`). This
automatically sets up a mail buffer with version information already
added. You just need to add a description of the problem, including a
reproducible test case and send the message.
